# Noninvasive Electrophysiological Biomarkers of Olfactory Responses Across Cognitive States in Alzheimer Dementia: Cross-Sectional Study

**DOI:** 10.2196/76245

**Published:** 2026-06-23

**Authors:** Juchan Ha, Junsoo Bok, Yunjin Lee, Yong Sung Kim, June Sic Kim, Hee-Jin Kim, Yongwoo Jang

**Affiliations:** 1 Department of Medical and Digital Engineering, College of Engineering, Hanyang University, 222, Wangsimni-ro, Seongdong-gu, Seoul, 04763, Republic of Korea, 82 2 2220 0655; 2 Department of Neurology, College of Medicine, Hanyang University, Seoul, 04763, Republic of Korea; 3 Clinical Research Institute, Konkuk University Medical Center, Seoul, 05030, Republic of Korea; 4 Department of Pharmacology, College of Medicine, Hanyang University, Seoul, 04763, Republic of Korea

**Keywords:** noninvasive olfactory recording, machine learning, digital biomarker, olfactory dysfunction, early diagnosis of dementia

## Abstract

**Background:**

Alzheimer disease (AD) is characterized by progressive cognitive decline, with olfactory dysfunction emerging among its earliest symptoms. Conventional olfactory tests rely on subjective self-reports and patient cooperation, limiting their clinical applicability. Noninvasive olfactory bulb (OB) recordings may provide objective physiological measures for early detection of cognitive decline.

**Objective:**

This study investigated the relationship between olfactory dysfunction and cognitive decline across the AD spectrum and evaluated whether noninvasive OB recordings obtained via a wearable electroencephalography-based device could serve as digital biomarkers for early detection and home-based monitoring.

**Methods:**

We recruited 71 participants, including individuals who were cognitively normal (CN; n=18), patients with mild cognitive impairment (MCI) subdivided into early MCI (n=30) and late MCI (n=9), and patients with dementia of the Alzheimer type (n=14). OB activity was recorded noninvasively during odor stimulation using a 6-channel wearable electroencephalography system. Behavioral olfactory function was assessed using a culturally adapted 6-item modified Brief Smell Identification Test and compared with electrophysiological responses. Time-frequency analyses identified disease-related components via nonparametric permutation testing (1000 iterations; *P*<.05). A support vector machine classifier was applied for group discrimination. Structural brain integrity was assessed through diffusion tensor imaging and magnetic resonance imaging, and cognitive performance was evaluated using comprehensive neuropsychological testing. Beta-band power (reflecting top-down cortical feedback) and gamma-band power (representing OB activity) were analyzed to characterize neural communication patterns.

**Results:**

Beta-band power progressively declined from CN to dementia of the Alzheimer type, while gamma-band power decreased and response latency increased beginning in late MCI. Behavioral olfactory testing failed to differentiate CN from early MCI (*P*=.77), whereas electrophysiological recordings revealed significant differences (*P*=.02). The support vector machine classifier achieved 83.1% accuracy in distinguishing all groups based on olfactory spectral features. Reduced beta-band power showed a trend-level association with cingulum-hippocampal tract diffusivity (*R*²=0.28; *P*=.061), whereas gamma-band power correlated with OB volume in later disease stages (*R*²=0.21; *P*<.001). Electrophysiological features explained 91.7% of the variance in cognitive scores (Seoul Neuropsychological Screening Battery II; *P*<.001). The brief 5 to 10-minute testing protocol, requiring less than 2 minutes to fit the device, supports its feasibility for repeated, home-based assessments.

**Conclusion:**

Bidirectional interactions between the OB and higher-order brain regions appear altered early in the AD continuum, with beta-band changes emerging first and gamma-band alterations occurring later. Digital OB recordings offer objective, noninvasive biomarkers for detecting cognitive decline before overt structural changes. Their portability and short duration make this approach ideal for longitudinal, home-based cognitive assessments within mobile health frameworks and for evaluating therapeutic efficacy in future interventional studies.

## Introduction

### Continuous Monitoring for Early Alzheimer Disease Detection

Alzheimer disease (AD) represents a complex clinical challenge characterized by gradual and heterogeneous neurodegenerative progression over years to decades. While AD pathology involves amyloid and tau accumulation, its clinical manifestations span a continuum from individuals who were cognitively normal (CN) with AD pathology to mild cognitive impairment (MCI) and dementia. The subtle and variable trajectory of cognitive decline limits the sensitivity of traditional, episodic clinical assessments such as positron emission tomography (PET) imaging or cerebrospinal fluid analysis [1,2]. Continuous and home-based monitoring technologies offer a transformative approach to early disease detection by enabling real-time tracking of subclinical functional changes in naturalistic settings [3-6]. Quantitative physiological and behavioral biomarkers, such as gait velocity, sleep architecture, and circadian activity, can reveal gradual deterioration over weekly or monthly timescales that are often missed during infrequent clinical evaluations. Integrating these continuous monitoring systems into clinical workflows expands the therapeutic window for both pharmacological and nonpharmacological interventions, including targeted cognitive rehabilitation and personalized lifestyle management [7].

Olfactory dysfunction is one of the earliest symptoms of AD. It is recognized both as an early indicator and a potential clinical marker of the progression and severity of the disorder [8-11]. In addition to cognitive impairment, approximately 90% of patients with dementia of the Alzheimer type (DAT) experience a loss of olfactory abilities, such as difficulties with olfactory recognition and identification [12-16]. These deficits were previously attributed to pathological changes in the olfactory bulb (OB) and associated regions, including the entorhinal cortex, hippocampal cornu ammonis-1 region, and subiculum [17-19]. However, the timing of olfactory dysfunction during the progression of AD and its relationship with cognitive function remain unclear. Sniffing kits, such as the University of Pennsylvania Smell Identification Test (UPSIT), are frequently used to examine olfactory dysfunction in patients suspected of having MCI or DAT [19-22]. Although behavioral olfactory tests such as UPSIT and the Brief Smell Identification Test (B-SIT) have demonstrated feasibility in cognitively impaired populations, they fundamentally depend on participants’ subjective reports of odor identification. This subjective nature introduces variability from individual interpretation, decision-making processes, and verbal or written communication abilities that may confound assessment of actual olfactory function [23-25]. Even with full participant cooperation, responses reflect a complex integration of sensory perception, cognitive processing, and motor output rather than isolated olfactory capability. In contrast, electrophysiological recordings from the OB capture objective neural signals during odor processing, providing quantifiable biomarkers in terms of latency, amplitude, and frequency components. Consequently, several studies have proposed using electroencephalography recordings during odor tests to reduce nonolfactory-related performance interference [26-30]. Structural magnetic resonance imaging (MRI) studies have also sought to differentiate MCI from DAT by evaluating volumetric decreases in the OB and primary olfactory cortex [31-34]. Therefore, olfactory evaluation using sniffing kits is likely to be undervalued in clinical assessment of AD despite its potential as an early indicator.

### Objectives

To address these challenges, recent studies have proposed noninvasive and temporally precise recordings of the human OB, validated across a range of experiments, as a reliable method for assessing signals from the OB [35,36]. This approach has demonstrated olfactory dysfunction in patients with Parkinson disease (PD) compared with participants who were CN [37]. In this study, we conducted noninvasive digital recordings of olfactory responses among individuals who were CN, patients with MCI, and patients with DAT, using advanced digital signal processing and machine learning algorithms to transform raw electrophysiological data into quantifiable biomarkers. This computational approach allows the extraction of subtle features from neural signals that can objectively differentiate between cognitive states. We compared the results of various clinical examinations, including diffusion tensor imaging (DTI), MRI, and neuropsychological assessments. This study aimed to (1) identify differences in olfactory responses between CN and patient groups to extract olfactory characteristics critical for AD diagnosis, (2) explore the association between olfactory response characteristics and cognitive impairment in clinically diagnosed MCI and DAT using a hospital-based cohort, and (3) establish how the dynamics of olfactory responses across frequency bands relate to structural brain changes observed through medical imaging. Digital recording of odorant-mediated OB responses addresses the subjectivity inherent in behavioral olfactory assessments by providing objective, reproducible neural measurements. Unlike self-reported odor identification, electrophysiological signals directly reflect the functional integrity of olfactory neural circuits and can reveal temporal dynamics of olfactory processing not accessible through behavioral testing alone. The rapid advancement of mobile health (mHealth) and ubiquitous health technologies has created new opportunities for continuous, home-based assessment of neurological function. Our electrophysiological olfactory testing approach embodies these paradigms by using a lightweight, wearable 6-electrode electroencephalography device that can be quickly applied by users or caregivers. The entire testing procedure requires only 5‐10 minutes, enabling regular self-assessment or caregiver-assisted monitoring in home settings. Unlike conventional clinic-based evaluations, this system allows individuals to track their olfactory and cognitive function through brief, repeatable sessions. Its portability and ease of use facilitate longitudinal monitoring, which is critical for detecting subtle, early changes in olfactory processing associated with neurodegeneration. We hypothesized that digital recordings of OB responses could provide a more comprehensive understanding of cognitive decline in AD, serving as a potential digital biomarker.

## Methods

### Study Population

This study used a cohort design across 3 cohorts: CN, MCI, and DAT. The prospective registry study of olfactory response heterogeneity in AD progression enrolled participants from 2 tertiary medical centers in the Republic of Korea (Hanyang University Hospital and Myongji Hospital) between September 2023 and September 2024. The community-dwelling cohort was recruited from 3 geographically diverse provinces: Seoul, Gyeonggi, and North Chungcheong. A total of 129 individuals were initially screened for this study, of whom 71 were included in the final analysis, including 18 CN controls, 39 individuals with MCI, and 14 patients with DAT. All participants with DAT satisfied the diagnostic criteria of the National Institute on Aging and the Alzheimer’s Association (NIA-AA) [25]. Comprehensive baseline assessments, including neuroimaging and neuropsychological evaluations, were conducted according to standardized protocols under physician supervision.

### Eligibility and Diagnostic Criteria

The study defined eligibility criteria for participant inclusion, restricting the age range for enrollment to 50‐85 years. Inclusion criteria required participants to have sufficient cognitive capacity to complete both neuropsychological and olfactory assessments. Participants with contraindications to neuroimaging were excluded. The diagnostic status was determined following the updated NIA-AA workgroup criteria [23]. Notably, MCI was diagnosed independently of the Seoul Neuropsychological Screening Battery II (SNSB-II) score using a comprehensive clinical evaluation. Diagnosis was based on documented cognitive decline corroborated by caregivers, objective episodic memory impairment on the delayed recall component of the Seoul Verbal Learning Test (performance below −1.5 SD, adjusted for age and education), a Clinical Dementia Rating score of 0.5, and fulfillment of the NIA-AA core clinical criteria for MCI due to AD. After the diagnosis of MCI was established, participants were further subdivided into early and late stages according to cognitive severity. The SNSB-II provides age-, sex-, and education-adjusted *z* scores across 5 cognitive domains (attention, language, visuospatial function, memory, and frontal and executive function), from which a composite *z* score was calculated by averaging domain scores. This composite score was used solely for severity stratification within the MCI group and was not involved in diagnostic determination. Participants with a composite *z* score ≥−1.0 were classified as early mild cognitive impairment (EMCI), whereas those with scores <−1.0 were classified as late MCI, in line with prior studies linking lower composite scores to broader multidomain impairment and increased risk of progression to dementia [38,39]. Using this criterion, 30 participants were classified as EMCI and 9 as late MCI. The relatively preserved composite scores observed in the EMCI group (mean 0.15, SD 0.58) reflect focal memory impairment that becomes attenuated when averaged across largely preserved cognitive domains, a pattern commonly observed in early-stage MCI. Clinical evaluation consisted of a comprehensive medical history review, neuroimaging analysis, and neuropsychological examination conducted by a qualified physician. Detailed diagnostic criteria are provided in Multimedia Appendix 1.

### Neuropsychological Assessments

Neuropsychological assessments were performed using a standardized neuropsychological battery called the SNSB-II [38]. This battery includes examinations of attention, language, calculation ability, visuospatial skills, memory, frontal-executive function, and general cognition, such as the Mini-Mental State Examination (MMSE) [40] and Clinical Dementia Rating. The SNSB-II comprises the digit span test (forward and backward), the Korean version of the Boston Naming Test, Rey-Osterrieth Complex Figure Test, Seoul Verbal Learning Test, Phonemic Controlled Oral Word Association Test, and Korean Color Word Stroop Test. All SNSB-II variables were converted to *z* scores and standardized for age, sex, and years of education.

### Amyloid PET and Structural Brain MRI

All participants underwent an 18F-florbetaben amyloid PET and structural MRI scan. Brain DTI was performed using a 3.0-T Achieva MRI system (Philips Medical Systems). DTI images were acquired in the transverse orientation using a pulse sequence with a single-shot spin-echo diffusion-sensitized echo-planar imaging sequence (15 gradient directions plus a B0 image with b value=800 s/mm², repetition time/echo time=13898/55 ms, field of view=224×224, matrix=112×112, number of averages=2, slice thickness=2.0 mm, and flip angle=90°). Amyloid positron emission tomography–computed tomography imaging was conducted following intravenous injection of 8 mCi of the amyloid radiotracer F-18 Florbetaben (Neuraceq; Piramal). Following a 90-minute uptake period, imaging was conducted for approximately 20 minutes using a dedicated PET/computed tomography (CT) scanner (Biograph 6; Siemens Medical Systems). The PET scans were reviewed by 2 expert PET readers and 1 neurologist based on predefined regional cortical tracer uptake and brain amyloid plaque load scoring systems. Four regions of interest, including the frontal, temporal, and parietal cortices along with the posterior cingulate and precuneus, were assessed in the visual analysis of the 18F-florbetaben PET scans. The regional cortical tracer uptake scores were condensed into a single 3-grade scoring system for each PET scan (brain amyloid plaque load score) as follows: 1 for no Amyloid-beta (Aβ) load, 2 for minor Aβ load, and 3 for significant Aβ load. Tract-based spatial statistics (TBSS) analysis was conducted to examine differences in DTI between patients with AD and healthy controls using the Functional Magnetic Resonance Imaging of the Brain Software Library (University of Oxford). The preprocessing pipeline included correction for head motion and eddy current distortions, followed by the computation of diffusion tensor metrics, specifically fractional anisotropy (FA), axial diffusivity, and radial diffusivity, to characterize the white matter fiber architecture. Subsequently, FA images were spatially normalized to a standardized brain template using the Functional Magnetic Resonance Imaging of the Brain nonlinear imaging registration tool. A mean FA skeleton was generated using projection techniques to represent the central white matter pathways common to all participants, with an applied threshold criterion of 0.2. Individual FA maps were then projected onto this skeleton for group-level analyses. Regional white matter tract identification was facilitated using the Johns Hopkins University ICBM-DTI-81 white matter atlas [41]. Statistical analyses used permutation-based nonparametric inference (5000 permutations) to assess between-group differences [42]. The statistical threshold was established at *P*<.05, with correction for multiple comparisons implemented using threshold-free cluster enhancement. Volumetric quantification of the OB was performed by manual delineation of sequential T1-weighted images using ImageJ (version 1.53h; National Institutes of Health). Bilateral OB annotations were independently conducted by 2 experienced raters using FreeView (Massachusetts General Hospital), a FreeSurfer visualization tool, and ambiguous cases were resolved by consultation with a qualified neurosurgeon [43]. To minimize bias, the raters were blinded to participant group assignment, age, and sex. Individual OB volumes were calculated by summing the product of the surface area measurements and slice thickness, with the total OB volume derived from the mean of the bilateral measurements.

### Behavioral Olfactory Testing

Olfactory function was assessed using a modified 6-item version adapted from the B-SIT, which itself is a 12-item subset of the 40-item UPSIT [44]. Participants were presented with a 4-category multiple-choice questionnaire and asked to identify which smells corresponded to the scent strip. As familiarity and responsiveness to certain odors may vary among ethnic groups, we selected 6 types of odors (cinnamon, smoke, chocolate, gasoline, soap, and onion) with a correct answer rate of 70% or more among healthy individuals from the 12 smells in B-SIT for this experiment. This culturally adapted version showed comparable diagnostic performance to the full B-SIT while avoiding misclassification due to odor unfamiliarity rather than true olfactory dysfunction.

### Digitalizing Olfactory Testing

**Figure 1.**
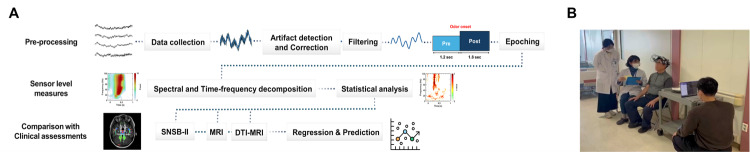
Experimental workflow for olfactory bulb signal analysis and clinical implementation. (A) Flow diagram illustrating the complete data processing pipeline: preprocessing of raw signals followed by data collection, artifact detection and correction, filtering, and epoching (with observation and rest periods of 3 seconds each). Secondary processing includes sensor-level measures with spectral and time-frequency decomposition, statistical analysis, and clinical comparisons using Seoul Neuropsychological Screening Battery II (SNSB-II), magnetic resonance imaging (MRI), and diffusion tensor imaging (DTI) with regression and prediction modeling. (B) Representative image of the experimental setup in a clinical environment, showing researchers administering the olfactory testing protocol to seated participants wearing the noninvasive olfactory bulb recording device.

The olfactory assessment protocol integrated the 6-item modified B-SIT, a validated and standardized instrument for evaluating olfactory function, with concurrent electrophysiological measurements to characterize the temporal dynamics of olfactory processing. Olfactory responses were recorded using a 6-channel active electrode electroencephalography system (Cyton; OpenBCI), and data were collected and digitized at a sampling rate of 250 Hz. In this study, the configuration of the OpenBCI electroencephalography cap was modified to optimize the detection of signals from the OB, following the approach proposed by Iravani et al [35]. Six electrodes were symmetrically placed on a mount along the centerline of the forehead, with each electrode spaced 5 cm apart to optimize the detection of OB signals. The helmet mount was designed to be adjustable, allowing the examiner to loosen it for easier electrode placement and subsequently tighten it to maintain firm and consistent contact with the participant’s skin. This configuration ensured reliable signal detection from the OB, as illustrated in Figures 1A and 1B. The electroencephalography signals were recorded continuously throughout the test session.

During olfactory testing, a respiratory sensor monitored participants’ breathing patterns to trigger olfactory stimulation. Recordings were segmented into epochs ranging from 1200 ms prestimulus to 1800 ms post stimulus, time locked to respiratory triggers, and rereferenced to the average of the left and right earlobe electrodes. Recorded signals were notch filtered at 60 Hz to eliminate power line noise. Additionally, muscle artifacts were detected by applying a bandpass filter (Butterworth filter, order 8) to the raw data, followed by a Hilbert transformation to extract the amplitude values. These values were standardized using *z* scores, with trials exceeding a *z* score of 6 assumed to be contaminated by muscle artifacts and excluded from further analysis. Considering the impact of eye movements and blinking on olfactory response signals, a lower *z* score threshold of 4 was used to improve the detection sensitivity, leading to the exclusion of trials with *z* scores exceeding this threshold. To further reduce blinking-related artifacts, participants were instructed to keep their eyes closed throughout the test. For signal processing, preprocessed and artifact-corrected olfactory signals were decomposed into time-frequency maps using a multitaper sliding window approach. This method was applied across a frequency range of 0.1‐100 Hz, with a step size of 0.1 Hz, and across a time range of –1 to 1 second, with a step size of 5 ms. Power in each time-frequency bin was estimated using 2 tapers derived from discrete prolate spheroidal sequences. To capture at least 2 cycles of each frequency bin, the taper length was selected to ensure accurate power estimation across the spectrum. Wavelet transformation for each time bin was conducted using 2 sets of wavelet functions derived from discrete prolate spheroidal sequence tapers. This transformation involved convolving the wavelet functions with the olfactory response signal in the frequency domain by multiplying the fast Fourier coefficients of the signal by those of the wavelets. The frequency-smoothing parameter was set to 80% of the value in each frequency bin. To account for motor responses to sniffing, baseline-corrected odor trials were compared with baseline-corrected sniff trials to analyze phase-locked responses, which were subsequently converted into decibels. For regression and classification, features were extracted by identifying time-frequency regions with maximal group differences via permutation testing and calculating each participant’s mean power within these regions. This process yielded 5 electrophysiological features in the beta and gamma bands for subsequent analyses. All preprocessing and time-frequency decompositions were performed using the open-source Fieldtrip toolbox in MATLAB (version R2023b; The MathWorks, Inc).

### Statistical Analysis

**Figure 2.**
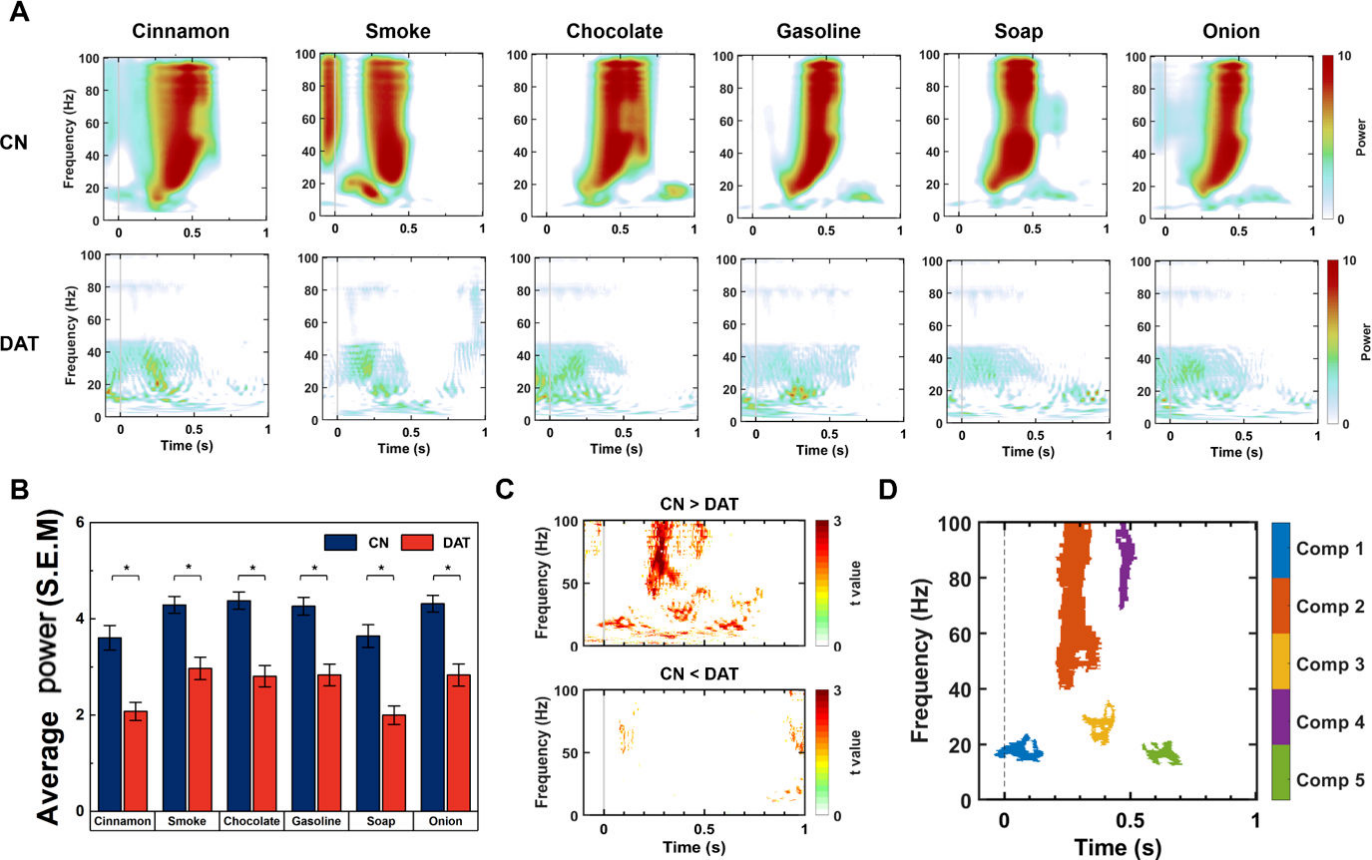
Odorant-mediated olfactory responses differentiate patients with dementia of the Alzheimer type (DAT) from cognitively normal (CN) controls. (A) Odor-mediated olfactory responses in CN controls and patients with DAT. Gamma synchronization was observed in CN controls following odor onset (indicated by the vertical gray line at time 0). In contrast, no synchronization was detected in the DAT group during the same period. (B) Averaged power change for odorant across the 0-1 second interval, presented with SE of the mean (SEM) for responses to the 6 selected odorants. Statistical significance is indicated by * for *P*<.05. (C) T-map derived from 1000 Monte Carlo permutation tests, illustrating significant differences in the beta (13‐30 Hz) and gamma (30-100 Hz) bands between the CN and DAT groups. Threshold t-maps indicate areas with higher power in CN controls compared to DAT at *P*<.05. (D) Threshold t-maps indicate significant clusters (*P*<.05; cluster size >300) that differentiate olfactory response components between the CN and DAT groups. Five distinct components were identified in the gamma and beta bands during early and late time points, with each component represented in a specific color. Color labels can be found in the color bar on the right side of the panel.

All statistical analyses were conducted using MATLAB (version R2023b) with the Signal Processing and Fieldtrip toolboxes [45]. Spectral densities of the 6 electrodes were averaged across the channels. Initially, the average power comparison was used to assess the intensity of gamma synchronization (30‐100 Hz) in the olfactory response one second after olfactory stimulation. Time-frequency maps of the olfactory responses between the 2 comparable groups were assessed using a nonparametric Monte Carlo permutation test. A total of 1000 permutation tests were conducted to identify significant clusters that differed between the 2 groups within the time-frequency maps, with significance defined as *P*<.05 and a cluster size exceeding 300 bins. This cluster-based permutation approach inherently controls for multiple comparisons across all time-frequency bins by evaluating cluster-level statistics, thereby maintaining the family-wise error rate without requiring additional correction methods. The identified clusters were labeled according to their latencies (for example, components 1 and 2; Figure 2). Machine learning classification was performed using a support vector machine with a radial basis function kernel. Model development involved leave-one-out cross-validation to maximize sample usage given the limited dataset. The 5 electrophysiological components were used as input features. Classification performance was evaluated using accuracy, precision, recall, and *F*_1_-score metrics. Regression and classification analyses used only the 5 electrophysiological components extracted from the time-frequency analysis as input features, without inclusion of demographic covariates. All *t* tests were 2-tailed. Statistical significance was indicated by asterisks, with * representing *P*<.05, ** denoting *P*<.01, and *** signifying *P*<.001.

### Ethical Considerations

This study was approved by the Institutional Review Board of Hanyang University Medical Center (2023-06-019) and conducted in accordance with the Declaration of Helsinki. All participants or their legally authorized representatives provided written informed consent and were informed of their right to withdraw at any time without penalty. All participant data were deidentified and assigned unique study codes, with the linking key stored separately in a password-protected file accessible only to the principal investigator. Neuroimaging data were anonymized using defacing algorithms to remove identifiable facial features. No financial compensation was provided to participants. However, participants received their complete diagnostic test results and medical consultations free of charge, which were disclosed as benefits of participation during the informed consent process.

## Results

### Overview

**Figure 3.**
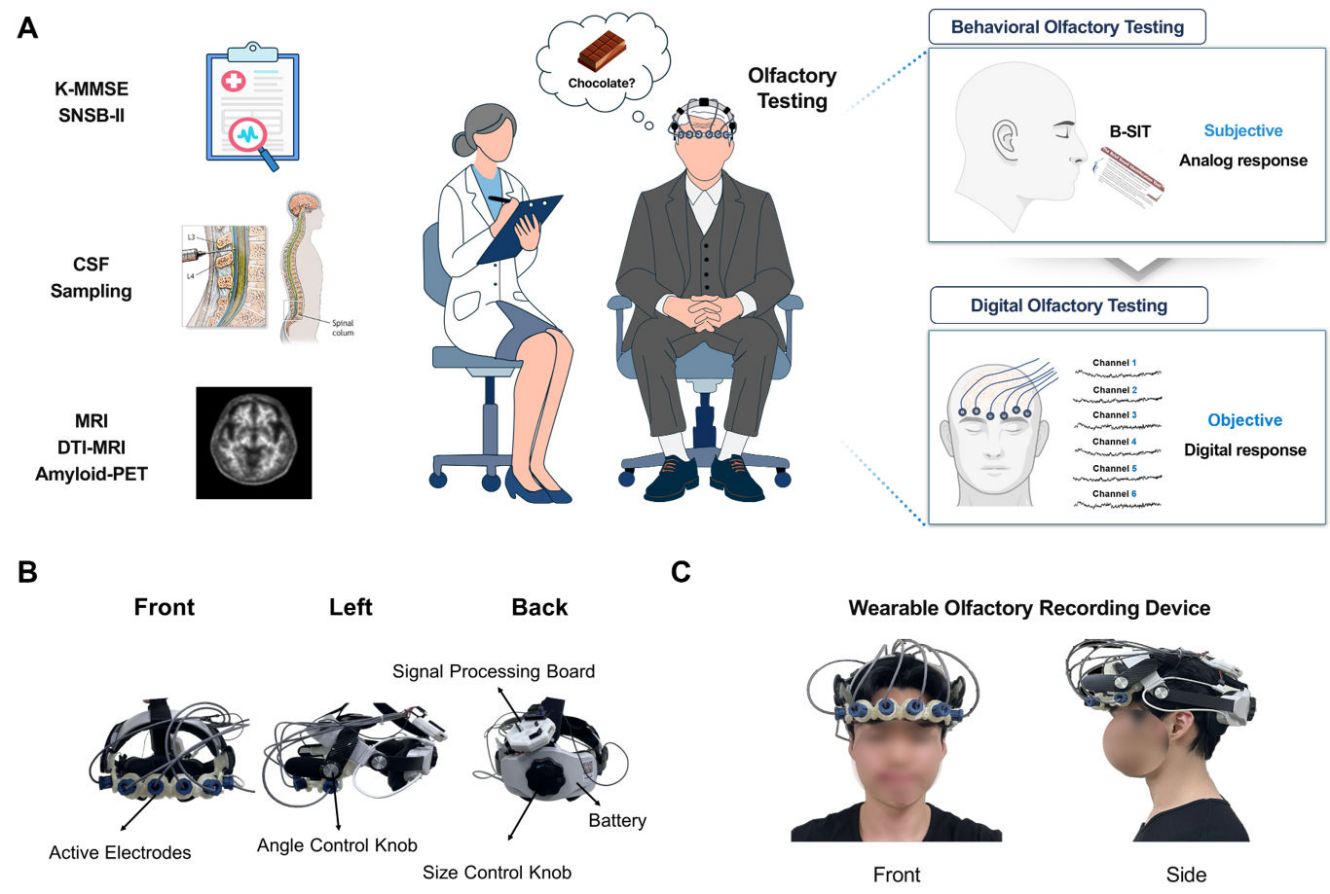
Overview of the methodological procedure for digital olfactory testing aimed at the early diagnosis of Alzheimer disease. (B) An illustration of the 6-electrode wearable olfactory response measurement appliance shown in three views (front, left, and back). (C) Noninvasive olfactory bulb signal measurement device in position. Front view showing the primary sensor array positioned over the nasal bridge with integrated electrodes for detecting olfactory neural activity. The device enables real-time acquisition of olfactory bulb signals without surgical intervention, facilitating neurophysiological monitoring. B-SIT: Brief Smell Identification Test; CSF: cerebrospinal fluid; DTI-MRI: diffusion tensor imaging–magnetic resonance imaging; K-MMSE: Korean Mini-Mental State Examination; MRI: magnetic resonance imaging; PET: positron emission tomography; SNSB-II: Seoul Neuropsychological Screening Battery II.

Digital olfactory testing was conducted alongside several traditional methods for AD diagnosis, including brain imaging techniques such as MRI and DTI, as well as neurobehavioral assessments such as the SNSB-II and MMSE. To evaluate olfactory ability, we conducted a 6-item modified B-SIT while simultaneously recording the electrical activity of the OB across the CN, MCI, andDAT groups. Demographic characteristics, cognitive status, and medication profiles of the participants are summarized in Table 1. Six electrodes were attached to positions capable of measuring OB responses for digital olfactory testing (Figure 3). The obtained signals were subjected to preprocessing and spectral analysis, resulting in a spectrogram. The spectral density of the signal was time-locked to stimulus onset and averaged across the 6 electrodes and trials to optimize the signal-to-noise ratio. The time-frequency map for the odor trials was determined within the specified time and frequency bands. To exclude contamination from sniffing and other motor-related artifacts, we compared the air and odor conditions. Odor event-related synchronization was observed in the beta (13‐30 Hz) and gamma (30‐100 Hz) bands at approximately 200‐550 ms post stimulus. Subsequently, specific areas showing significant differences between the CN and DAT groups were identified using nonparametric tests with 1000 permutations, resulting in the isolation of 5 distinct components. The 5 extracted components were analyzed for correlations with existing medical imaging results, such as MRI and DTI findings, as well as neurobehavioral test outcomes (MMSE and SNSB-II), to validate their effectiveness. This process was investigated to determine whether these olfactory characteristics could serve as early diagnostic markers of AD. An illustration of this process is shown in Figure 1.

**Table 1.** Demographic and clinical characteristics according to diagnostic group. Values are presented as mean (SD) or n (%). *P* values are from post hoc pairwise comparisons with the cognitively normal (CN) group using Bonferroni correction. Overall group differences (one-way ANOVA or chi-square test) were as follows: age (*F*_3,67_=3.21; *P*=.03), sex (*χ*²_3_=3.84; *P*=.28), Brief Smell Identification Test (*F*_3,67_=8.45; *P*<.001), 6-item modified Brief Smell Identification Test (*F*_3,67_=9.72; *P*<.001), education (*F*_3,67_=2.89; *P*=.04), Mini-Mental State Examination (*F*_3,67_=15.32; *P*<.001), Seoul Neuropsychological Screening Battery II (*F*_3,67_=12.84; *P*<.001), Clinical Dementia Rating (Kruskal-Wallis H test statistic [H_3_]=21.45; *P*<.001), Clinical Dementia Rating–Sum of Boxes (H_3_=28.93; *P*<.001), brain amyloid plaque load (*F*_3,67_=6.78; *P*<.001), and APOE ε4 (*χ*²_3_=7.92; *P*=.05).

Characteristic	CN^a^ (n=18)	EMCI^b^ (n=30)	Late MCI (n=9)	DAT^c^ (n=14)	*P* value vs CN (EMCI/LMCI^d^/DAT)
Age (years), mean (SD)	72.17 (6.37)	70.87 (6.97)	70.67 (6.88)	76.79 (4.14)	.65/.62/.02
Sex (female), n (%)	15 (83)	20 (66)	6 (66)	8 (57)	.18/.27/.09
B-SIT^e^ score, mean (SD)	7.94 (2.09)	8.27 (2.03)	5.78 (2.90)	4.79 (2.37)	.54/.02/<.001
6-SIT^f^, mean (SD)	4.89 (0.99)	4.8 (1.24)	3.33 (1.83)	2.71 (1.91)	.77/.02/<.001
Education years, mean (SD)	10.06 (5.16)	9.63 (5.34)	11.22 (3.58)	6.07 (4.95)	.76/.58/.02
MMSE^g^, mean (SD)	27.78 (1.96)	26.67 (2.94)	23.78 (2.70)	19.79 (3.30)	.14/<.001/<.001
SNSB-II^h^, mean (SD)	54.38 (5.75)	51.33 (7.53)	44.62 (5.29)	32.76 (5.01)	.12/.004/<.001
CDR^i^, mean (SD)	0.42 (0.19)	0.43 (0.17)	0.5 (0.00)	0.93 (0.37)	.86/.08/<.001
CDR-SB^j^, mean (SD)	0.69 (0.50)	0.90 (0.92)	2.28 (1.08)	5.61 (2.04)	.37/.003/<.001
BAPL^k^, mean (SD)	1.13 (0.85)	1.77 (0.88)	2.22 (0.92)	2.64 (0.72)	.06/.03/.002
APOE4^l^ ε4 carrier, n (%)	8 (44)	12 (40)	7 (77)	10 (71)	.76/.04/.03

a CN: cognitively normal.

b EMCI: early mild cognitive impairment.

c DAT: dementia of the Alzheimer type.

d LMCI: late mild cognitive impairment.

e B-SIT: Brief Smell Identification Test.

f 6-SIT: 6-item modified Smell Identification Test.

g MMSE: Mini-Mental State Examination.

h SNSB-II: Seoul Neuropsychological Screening Battery II.

i CDR: Clinical Dementia Rating.

j SB: sum of boxes.

k BAPL: brain amyloid plaque load.

l APOE: apolipoprotein E.

### Component Identification Through CN-AD Contrast Analysis

**Figure 4.**
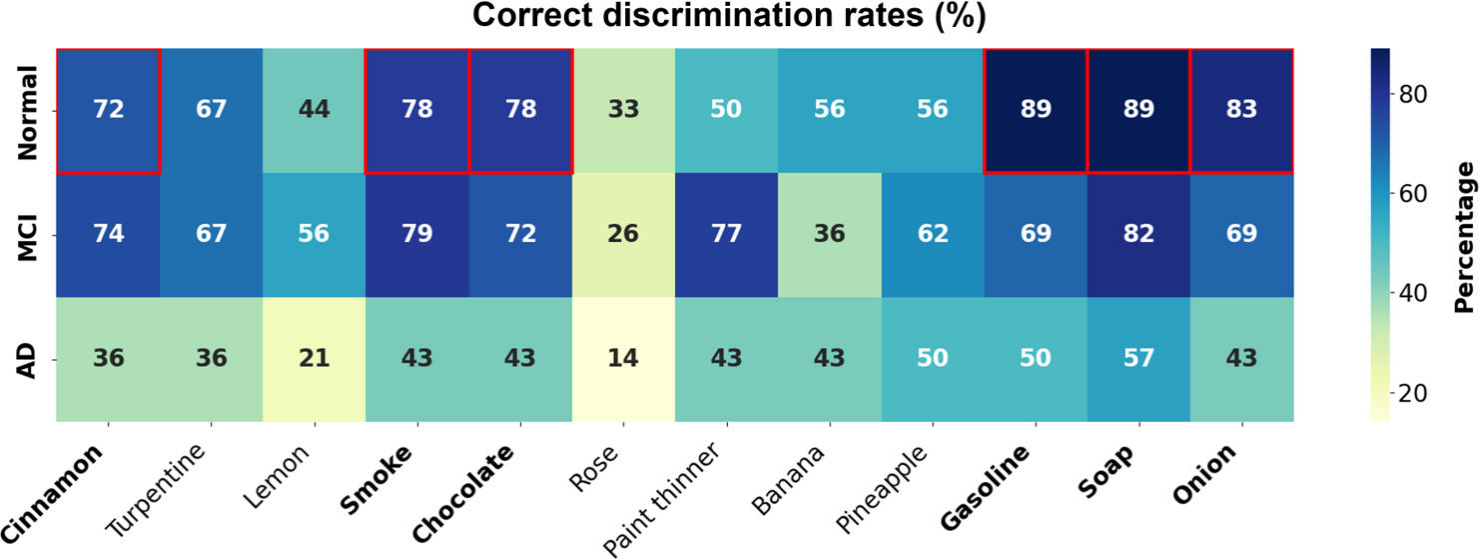
Rates of correct answers for each Brief Smell Identification Test odor identification question, categorized into 3 patient groups. The shading of each bin indicates the rates of correct answers, with darker colors representing higher accuracy and lighter colors indicating higher error rates. Odors that achieved over 70% correct answers in the cognitively normal group are highlighted with a red border and marked in bold in the labels below. CN: cognitively normal; DAT: dementia of the Alzheimer type; MCI: mild cognitive impairment.

**Figure 5.**
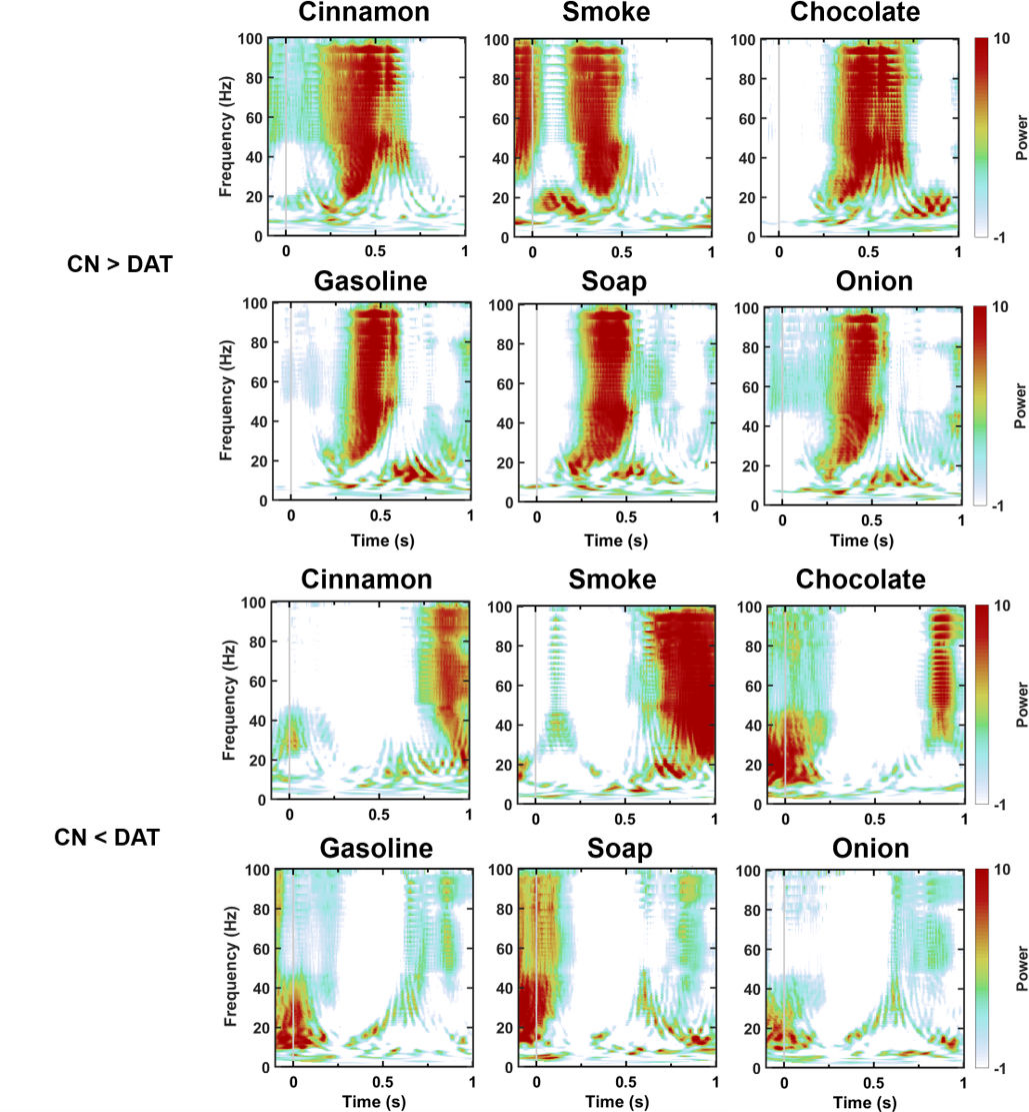
Time-frequency representation of odor-mediated olfactory responses for each selected odorant, shown as a 3 × 2 grid of the six odorants (cinnamon, smoke, and chocolate in the top row; gasoline, soap, and onion in the bottom row). The upper block (CN > DAT) indicates higher power for the cognitively normal (CN) group relative to the dementia of Alzheimer type (DAT) group, and the lower block (CN < DAT) indicates lower power for the CN group relative to the DAT group. Gamma synchronization is observed in the normal controls after odor onset, as indicated by the vertical gray line at time 0.

Patients with DAT reported olfactory impairment when assessed using the B-SIT [20,22,25], which included 12 odors selected from the UPSIT [44]. As familiarity and responsiveness to certain odors may vary among ethnic groups, we first selected 6 types of odors (cinnamon, smoke, chocolate, gasoline, soap, and onion) with a correct answer rate of 70% or more among healthy individuals from the 12 smells in the B-SIT (Figure 4). Behavioral olfactory testing failed to distinguish between the CN and MCI groups. Discrimination rates were comparable across all 6 odors tested, with both groups showing relatively preserved identification performance (mean correct: CN 81.5%, SD 16.5% vs MCI 74.2%, SD 25.1%; *P*=.14). Only the clinical dementia group showed significant impairment (mean 45.3%, SD 31.8%; *P*<.001 vs both CN and MCI). Figure 2A shows the spectrum of olfactory responses to the 6 selected odors in healthy individuals and patients with DAT. In the CN group, odor-responsive signals were observed within 0.6 seconds after odor exposure, specifically in the beta and gamma frequency bands across 6 electrodes. Conversely, patients with DAT showed no detectable gamma synchronization at these temporal and frequency intervals, thereby confirming the ability of the control group to identify robust olfactory signals. As shown in Figure 2B, the average power recorded in the response of patients with DAT to the selected odors was significantly lower than that in the CN group for cinnamon (*P*=.02), smoke (*P*=.04), chocolate (*P*=.01), gasoline (*P*=.03), soap (*P*=.01), and onions (*P*=.02). This uniform impairment across chemically diverse odorants indicates a central olfactory processing deficit rather than peripheral receptor-specific dysfunction. Figure 2C shows the statistically significant differences in odor responses observed between the CN and DAT groups. To identify specific regions between the 2 groups, we performed nonparametric tests with 1000 permutations. The results are indicated by threshold t-maps, highlighting areas with *P*<.05. The upper panel demonstrates that the CN group showed enhanced power in the beta and gamma bands in response to the selected odors compared to the DAT group (CN > DAT). Notably, no significant difference in power was observed in patients with DAT when compared to the CN group (CN < DAT). This analysis revealed significant increases in the beta and gamma band power in the CN group compared to the DAT group at approximately 250 ms postodor onset (*t*_30_=2.1192; *P*=.02). Conversely, a decline in gamma frequency power was observed at approximately 950 ms and 980 ms in the CN group compared with the DAT group (*t*_30_=−1.8412; *P*=.04). Significant clusters (*P*<.05; cluster size > 300) were identified and sequentially labeled based on their temporal order as component 1, component 2, and so on. As shown in Figure 2D, 5 distinct components were identified, each of which was visually represented by a different color. The statistical parameters for each component were as follows: component 1 (*t*_30_=1.9934; *P*=.03), component 2 (*t*_30_=2.1823; *P*=.03), component 3 (*t*_30_=1.9938; *P*=.03), component 4 (*t*_30_=1.9182; *P*=.04), and component 5 (*t*_30_=2.1393; *P*=.02). Additionally, Figure 5 provides the differences in the time-frequency maps between the CN and DAT groups in response to each of the 6 odorants.

### Stage-Dependent Spectral Alterations and Clinical Associations

**Figure 6.**
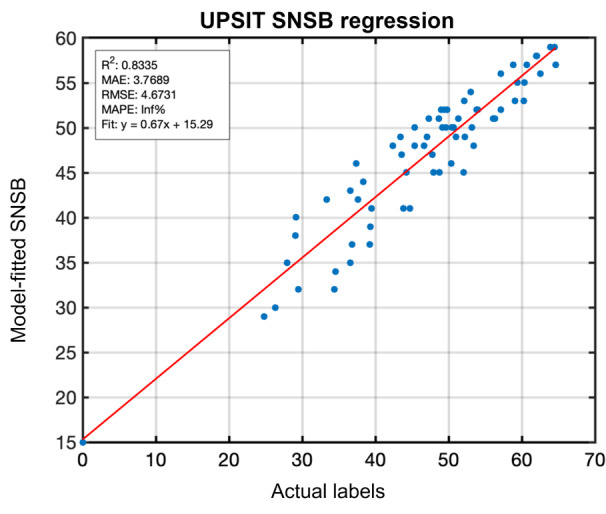
Plot of model-fitted Seoul Neuropsychological Screening Battery II (SNSB-II) scores with University of Pennsylvania Smell Identification Test (UPSIT) vs actual SNSB-II scores using the multiple linear regression model, with the selected 6 UPSIT scores as the independent variables and the SNSB-II score as the dependent variable. The mean absolute error (MAE), root mean square error (RMSE), and model fit are expressed as the adjusted *R*^2^ of the model.

**Figure 7.**
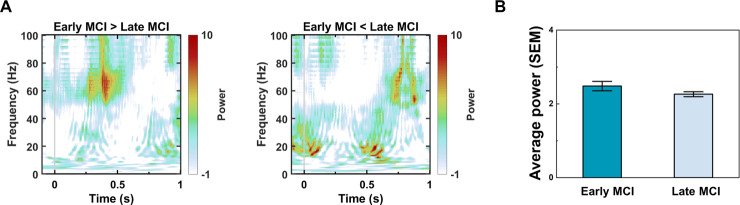
Averaged time-frequency map of 6 odorant-mediated olfactory responses, demonstrating higher power in the early mild cognitive impairment (MCI) group compared to the late MCI group, and averaged odorant-mediated olfactory responses showing reduced power in the late MCI group compared to the early MCI group. Warmer colors in the time-frequency response represent higher power values, as shown in panel A. Averaged power change for odorants across the 0-1 second interval, presented with SE of the mean (SEM) for responses to the 6 selected odorants, as shown in panel B. Statistical significance is indicated by * for *P*<.05.

**Figure 8.**
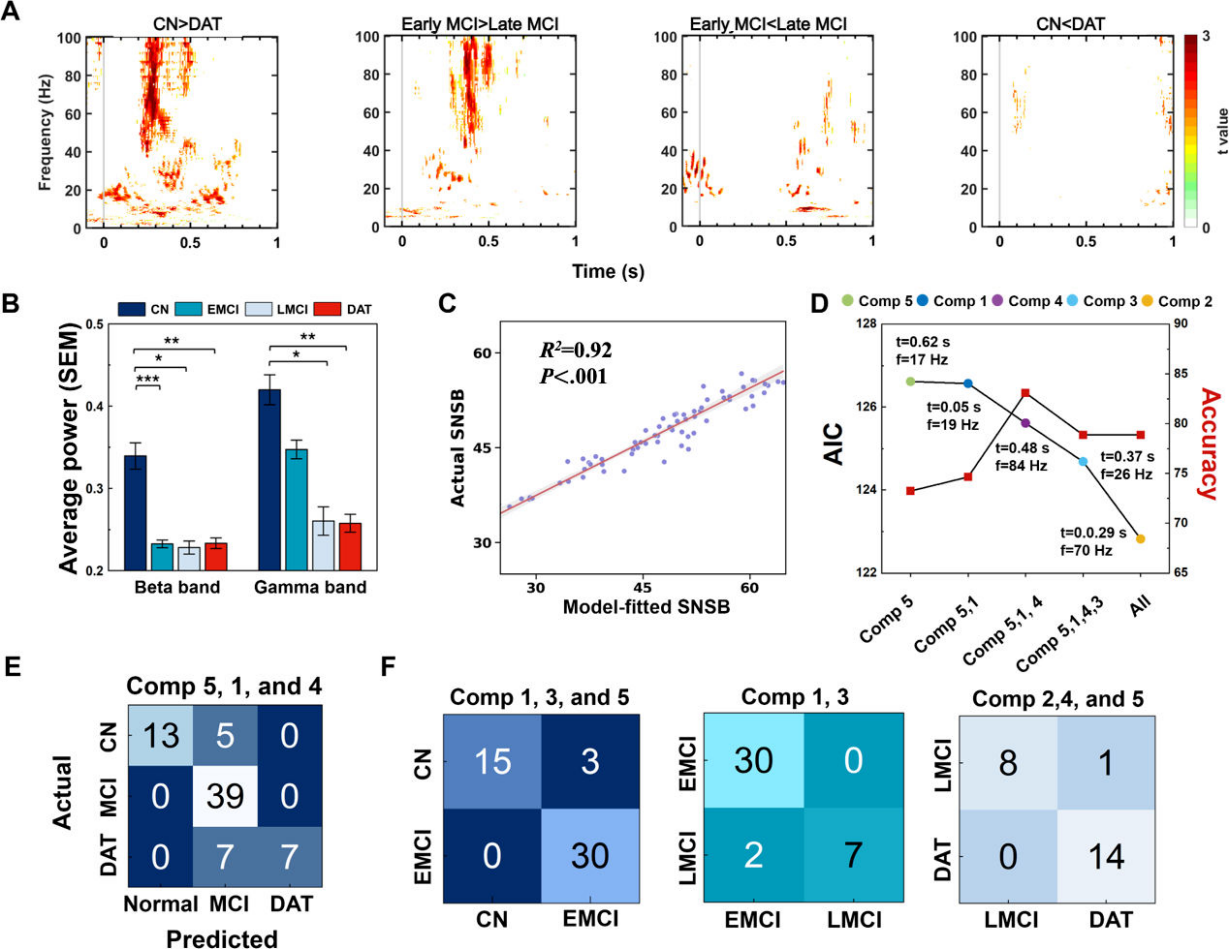
Olfactory response components as a digitalized diagnosis tool. (A) Threshold t-maps derived from 1000 Monte Carlo permutation tests at *P*<.05, indicating a gradual decrease in gamma synchronization and delayed reaction time from individuals who were cognitively normal (CN) to patients with dementia of the Alzheimer type (DAT), significant in the beta and gamma bands. (B) Averaged power change for odorants from each olfactory component, presented with SE of the mean (SEM) for responses to the 6 selected odorants (components 1, 3, and 5 exhibited the beta band, whereas components 2 and 4 belonged to the gamma band). Statistical significance was indicated using asterisks, with * representing *P*<.05, ** denoting *P*<.01, and *** signifying *P*<.001. (C) Plot of the predicted Seoul Neuropsychological Screening Battery II (SNSB-II) scores vs actual SNSB-II scores using the multiple linear regression model, regressing the extracted key features from the olfactory response components as dependent variables. The model fit is expressed as the adjusted *R*^2^ of the model and the Pearson correlation between the 2 variables, indicating the proportion of variance in SNSB-II scores explained by the model parameters. (D) Akaike information criteria (AIC) for each olfactory response component. Accuracy of models with stepwise adding of components shows maximum accuracy for the model, including components 5, 1, and 4. (E) Confusion matrix of the support vector machine classifier model: out of 18 individuals in the CN group, 13 were classified correctly as CN, whereas 5 were misclassified as mild cognitive impairment (MCI). The classification was accurate for the MCI groups. Among 14 individuals in the DAT group, 7 were misclassified as MCI. (F) Cross-tables distinguishing adjacent cognitive stages (cognitively normal vs early mild cognitive impairment, early vs late mild cognitive impairment, and late mild cognitive impairment vs dementia of the Alzheimer type) show true-positive, false-positive, true-negative, and false-negative values. Comp: component; EMCI: early mild cognitive impairment; LMCI: late mild cognitive impairment.

**Figure 9.**
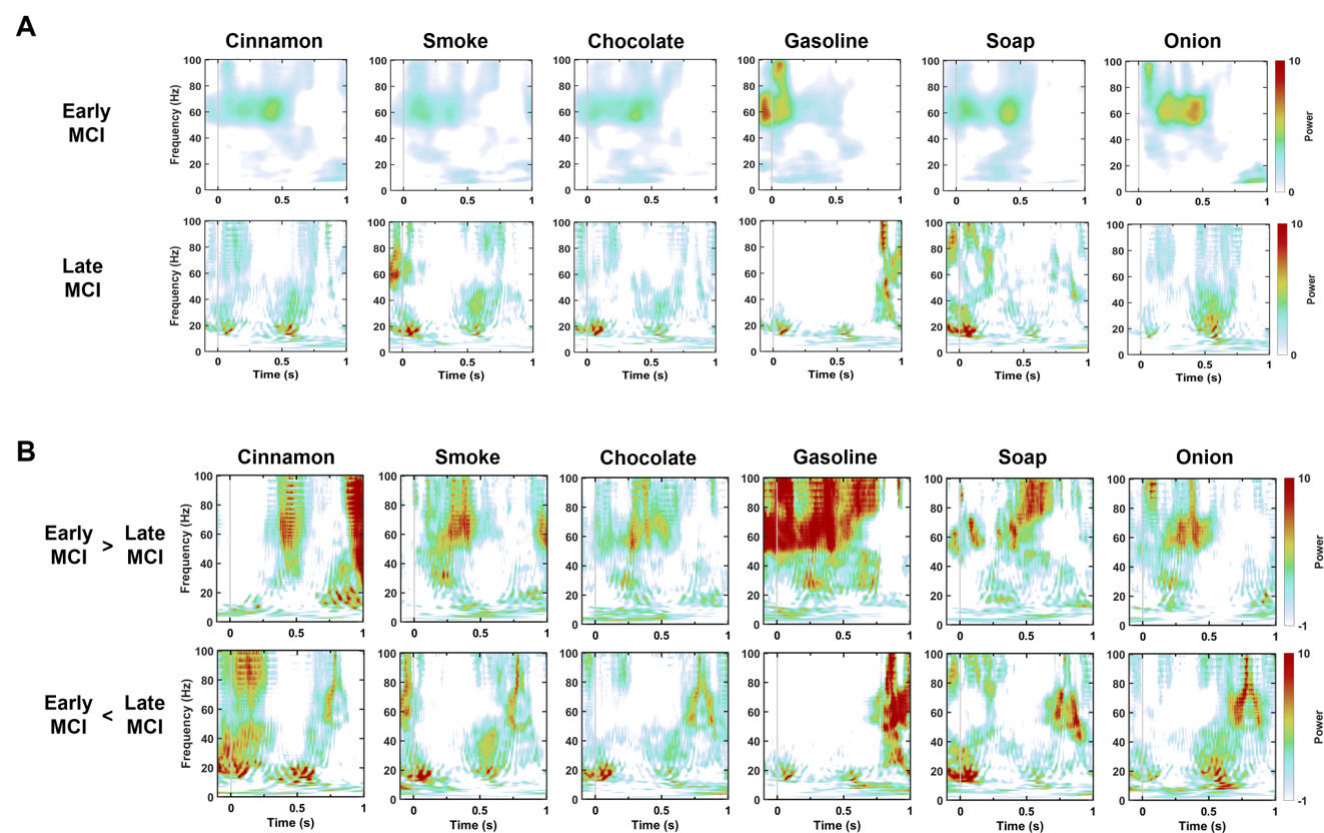
Time-frequency representation of odor-mediated olfactory responses in early and late mild cognitive impairment (MCI) for each of the 6 selected odorants (cinnamon, smoke, chocolate, gasoline, soap, and onion), arranged in columns. (A) Absolute time-frequency responses for the early MCI group (top row) and the late MCI group (bottom row). Early gamma synchronization was observed in the early MCI group shortly after odor onset (indicated by the vertical gray line at time 0), whereas synchronization was delayed in the late MCI group. (B) Between-group contrasts: the top row (early MCI > late MCI) indicates higher power in the early MCI group, and the bottom row (early MCI < late MCI) indicates higher power in the late MCI group. Comparably high gamma synchronization was observed in both MCI groups after odor onset, in contrast to the markedly reduced responses previously shown for the dementia of the Alzheimer type group.

**Figure 10.**
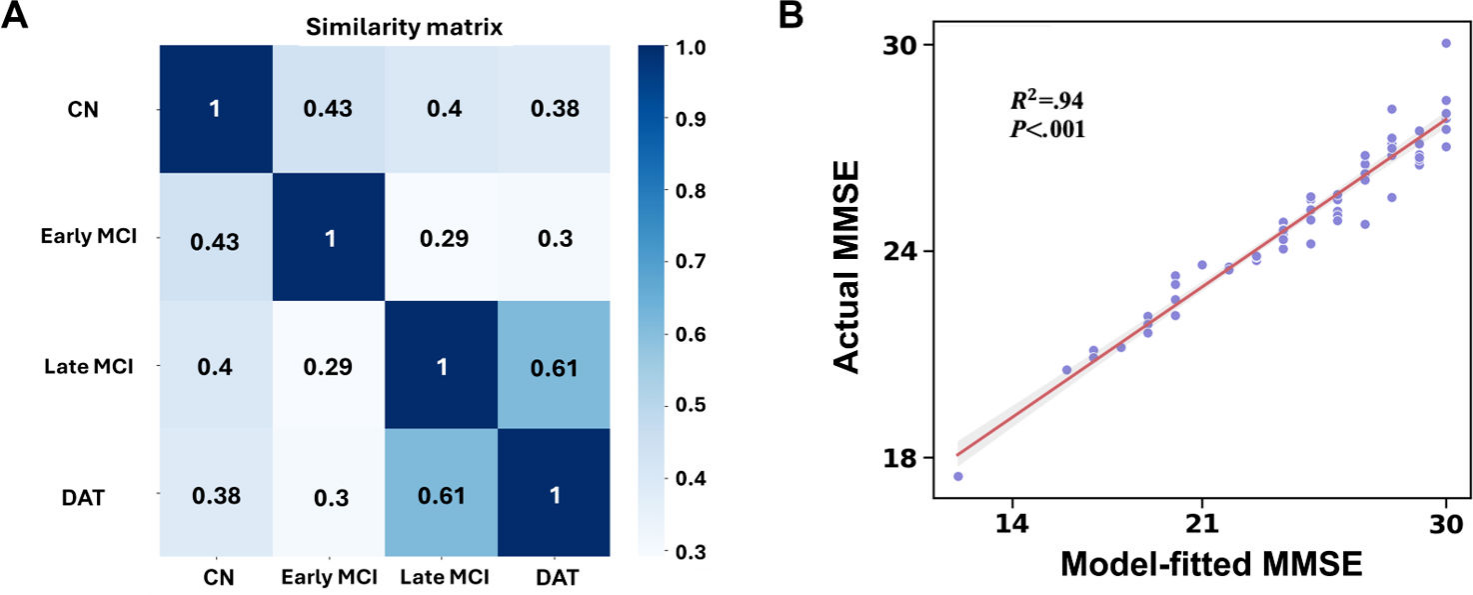
Structural similarity index matrix (SSIM) of olfactory response spectrograms across the cognitively normal (CN), early mild cognitive impairment (MCI), late MCI, and dementia of the Alzheimer type (DAT) groups. Higher values indicate greater similarity between spectrograms; high similarity was observed between the CN and early MCI groups (SSIM=0.43) and between the late MCI and DAT groups (SSIM=0.61), as shown in panel A. Plot of predicted vs actual Mini-Mental State Examination (MMSE) scores using the multiple linear regression model, with the extracted key features from the olfactory responses as the independent variables and the MMSE score as the dependent variable, as shown in panel B. The model fit is expressed as the adjusted *R*² and the Pearson correlation between the 2 variables, indicating the proportion of variance in MMSE scores explained by the model parameters. The shaded band denotes the 95% CI of the regression line.

For the loss of smell to be used as an early diagnostic method for AD, the detection of early signs is essential during the MCI stage, which precedes dementia. However, existing approaches do not clearly differentiate between individuals who were CN and those with MCI. Therefore, we conducted an experiment to determine whether digitalized measurements of olfactory function could effectively classify MCI severity. To examine the progression of olfactory dysfunction across the cognitive spectrum, the MCI group was stratified according to cognitive severity. Using the SNSB-II composite *z* score with a cutoff of –1.0 (adjusted for age and education), 30 participants were identified as EMCI (mean *z* score 0.15, SD 0.58) and 9 as late MCI (mean *z* score –1.65, SD 0.42) [38,39]. This score reflects multiple cognitive domains, including language, attention, visuospatial skills, memory, and frontal and executive function. Neuropsychological evaluations and relationships between the groups are summarized in Table 1 and Figure 6. First, we generated a differential time-frequency spectrum comparing EMCI to late MCI (EMCI > late MCI) and vice versa (EMCI < late LMCI). No significant differences in the odor-induced gamma band power were observed among the groups. Notably, the EMCI group exhibited faster responses within 490 ms of odor onset across the 6 electrodes. In contrast, the late MCI group showed delayed gamma synchronization at approximately 850 ms post stimulus after controlling for sniffing and evoked responses (Figure 7A). However, no difference in the average gamma-band power was found between the groups (Figure 7B). Furthermore, we analyzed significant increases in the average power spectrum across the CN, EMCI, late MCI, and DAT groups. As shown in Figure 8A, the gamma band power spectrum showed a significant reduction and delayed response time starting from the late MCI stage compared with individuals who were CN. Additionally, the beta band power spectrum progressively decreased as the condition advanced from CN to EMCI, LMCI, and DAT stages. These results were consistent for each of the 6 selected odors, as shown in Figures 9A and 9B. In this regard, the structural similarity index matrix (SSIM) analysis of the olfactory response spectrograms revealed high similarity between the control and EMCI groups (SSIM=0.43) and between the late MCI and DAT groups (SSIM=0.61), as provided in Figure 10. Olfactory characteristics were derived by computing the mean values across the temporal stages for each component. Components 1, 3, and 5 were associated with the beta band, whereas components 2 and 4 were associated with the gamma band (Figure 2D). The spectral analysis in Figure 8B revealed significant decreases in the beta band power from EMCI (*P*<.001) to late mild cognitive impairment (LMCI; *P*=.03), culminating in DAT (*P*=.009). The gamma band power demonstrated a distinct temporal progression, with significant reductions emerging at LMCI (*P*=.01) and progressing through DAT (*P*=.001), while showing marginal alterations at EMCI (*P*=.08).

To evaluate clinical applicability, the extracted spectral characteristics, categorized into beta and gamma frequency bands, were used to examine their association with cognitive performance. As shown in Figure 8C, multiple linear regression analysis demonstrated that electrophysiological features explained 91.69% of the variance in SNSB-II *z* scores (*P*<.001). This remarkably strong association between olfactory biomarkers and cognitive function was consistent across the entire cognitive spectrum from CN to DAT. The extremely low *P* value indicates that this relationship is highly unlikely to have occurred by chance. Additionally, MMSE scores showed similarly strong associations with olfactory responses (*R*^²^=0.94; Figure 10). These results demonstrate that the magnitude of electrophysiological responses in beta and gamma bands closely tracks with the severity of cognitive impairment, supporting the potential of these biomarkers for objective cognitive assessment.

Model selection was conducted using the Akaike information criterion to assess the accuracy of fit for each olfactory component based on the in-sample prediction error. In classifying the CN, MCI, and DAT groups, component 2 showed the optimal performance, with the lowest Akaike information criterion value of 122.82, followed by component 3 (124.69), component 4 (125.61), component 1 (126.57), and component 5 (126.62). Subsequently, we used a stepwise component addition approach within the regression framework to optimize the prediction accuracy. The analysis revealed a peak performance with a 3-component model that included components 5, 1, and 4, as shown in Figure 8D. We implemented a support vector machine regressor that integrates component-specific olfactory characteristics to assess the diagnostic utility of digitalized olfactory response recordings. Model performance was evaluated using leave-one-out cross-validation, where each participant was held out as a test sample. The resulting classification model demonstrated exceptional discriminative capability across the 3 groups, achieving 83.09% accuracy, 88.64% precision, perfect recall (100%), and an *F*_1_-score of 93.98% (Figure 8E). These metrics underscore the robust diagnostic potential of the proposed multicomponent approach. Therefore, the strong predictive relationship between odorant-mediated olfactory responses and cognitive performance underscores the potential clinical utility of this method for early diagnosis of AD. Further analyses were performed to evaluate the component-specific contributions in distinguishing between adjacent cognitive stages (CN-EMCI, EMCI-LMCI, and LMCI-DAT) to identify the key components for the early detection of AD. The discrimination between CN and EMCI states was achieved with remarkable precision using components 1, 3, and 5, all representing beta band activity, resulting in 93.75% accuracy, 90.91% precision, and 100% recall (Figure 8F). Notably, the predominant use of beta band components rather than gamma band components in the pre-DAT stage classification aligned with the findings presented in Figure 8B. The gamma band components were critical in differentiating patients with DAT from the LMCI group, marking a distinct transition point in disease progression. Table 2 summarizes the classification performance across different diagnostic comparisons.

**Table 2.** Classification performance of support vector machine models using olfactory electrophysiological features. Performance metrics for support vector machine classification across different diagnostic group comparisons. Models were trained using electrophysiological components extracted from time-frequency analysis of olfactory responses. Leave-one-out cross-validation was performed for the 3-way classification. Binary classifications between adjacent cognitive stages reveal differential importance of frequency bands: beta-band components (1, 3, and 5) were most effective for distinguishing cognitively normal from early MCI, whereas gamma-band components (2 and 4) became crucial for late-stage discrimination.

Classification task	Accuracy (%)	Precision (%)	Recall (%)	*F*_1_-score (%)	Features
3-way classification (CN^a^, MCI^b^, and DAT^c^)	83.09	88.64	100	93.98	5,1,4
CN vs EMCI^d^	93.75	90.91	100	95.24	1,3,5
EMCI vs LMCI^e^	87.18	85.71	92.31	88.89	1,3
LMCI vs DAT	82.61	88.89	88.89	88.89	2,4,5

a CN: cognitively normal.

b MCI: mild cognitive impairment.

c DAT: dementia of the Alzheimer type.

d EMCI: early mild cognitive impairment.

e LMCI: late mild cognitive impairment.

### Structural Correlations of Bidirectional Spectral Components

**Figure 11.**
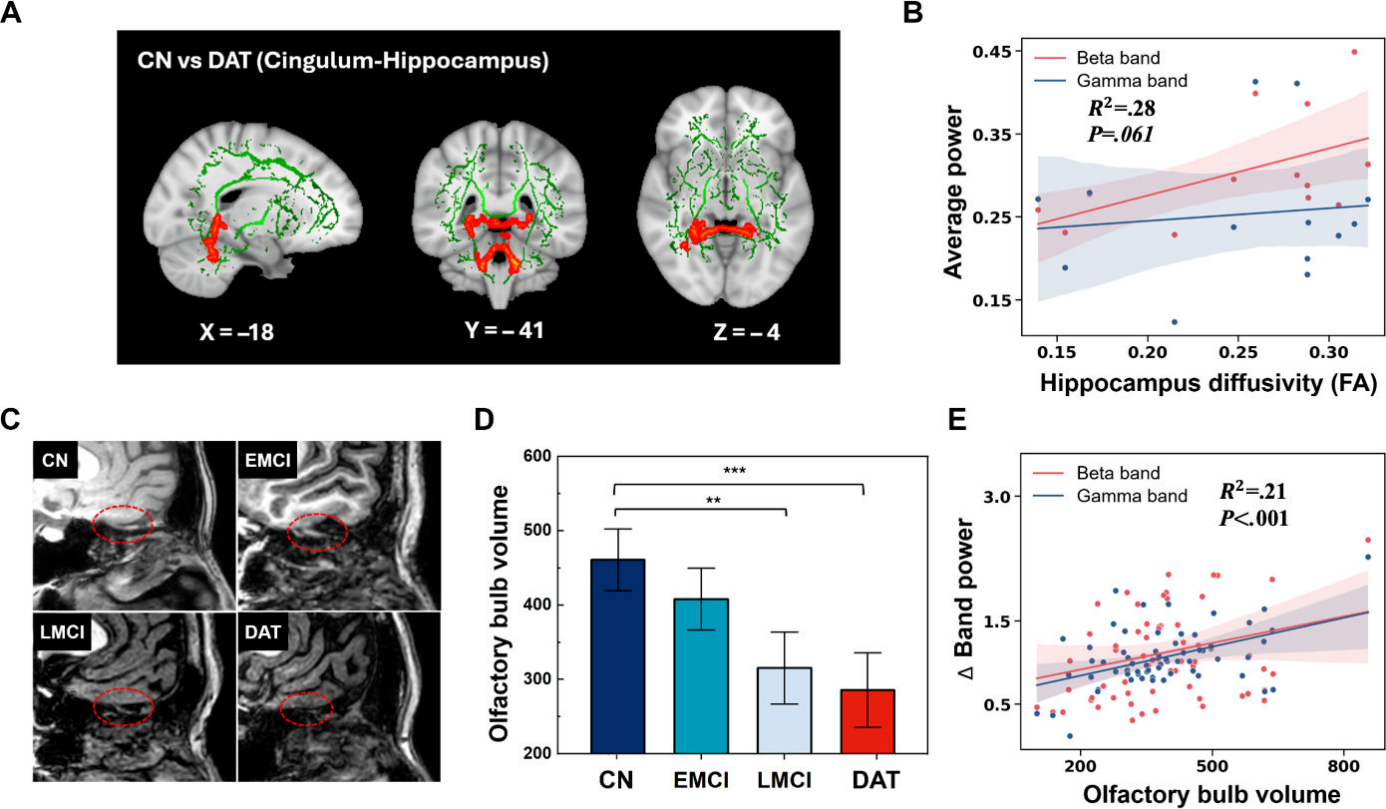
Comparison of olfactory responses with clinically relevant structural evidence. (A) White matter structures showing a significant fractional anisotropy (FA) decrease in the hippocampal cingulum in the dementia of the Alzheimer type (DAT) group (*P*<.05). Statistical images were overlaid onto the mean skeleton (green) and the MNI152 template (gray scale) for visualization (B) FA values of the hippocampal cingulum positively correlated with the beta-band olfactory component (mean of components 1, 3, and 5), whereas gamma-band components (components 2 and 4) exhibited minimal correlation with hippocampal white matter integrity. (C) Representative T1-weighted magnetic resonance imaging of each cohort (cognitively normal [CN], early mild cognitive impairment, late mild cognitive impairment, and DAT, from left to right) with OB regions delineated by red dashed circles. (D) Olfactory bulb volume change across CN to DAT, presented with SE of the mean (SEM). Statistical significance was indicated using asterisks, with * representing *P*<.05, ** denoting *P*<.01, and *** signifying *P*<.001. (E) The olfactory bulb volume positively correlated with the gamma-band olfactory component (mean of components 2 and 4), whereas beta-band components (components 1, 3, and 5) exhibited minimal optimization in linear fitting. EMCI: early mild cognitive impairment; LMCI: late mild cognitive impairment.

To elucidate the brain areas associated with the beta and gamma components within the olfactory system, we used a dual analytical approach that combined TBSS and volumetric MRI quantification. TBSS analysis revealed significantly reduced FA in the hippocampus cingulum of the DAT group compared with that in the CN group (*P*<.05), as shown in Figure 11A. The inverse correlation between FA values and cognitive impairment suggests that reduced white matter integrity in the hippocampal cingulum region is associated with more severe cognitive deficits [46-48]. Further analysis revealed a positive correlation approaching statistical significance between the beta-band components (mean of components 1, 3, and 5) in olfactory responses and FA values within the hippocampal-cingulum tracts in patients with DAT (*R*^²^=0.28; *P*=.06), consistent with beta-band oscillations representing top-down cortical feedback signals. In contrast, gamma-band components showed minimal correlation with the integrity of the hippocampal-cingulum tracts (*R*^²^=0.013; *P*=.53; Figure 11B).

In T1-weighted MR images, volumetric analysis of the OB revealed progressive atrophy across the disease spectrum, with a notable reduction in volume as cognitive status declined from CN to EMCI, LMCI, and DAT stages. Figure 11C provides representative T1-weighted MR images from each stage, with the OB regions delineated by red dashed circles, illustrating sequential volume reduction corresponding to cognitive decline. Quantitative analysis revealed significantly reduced OB volumes in patients with LMCI compared with those in the CN group (*P*=.005), whereas patients with DAT exhibiting a pronounced volume reduction (*P*<.001), as shown in Figure 11D. In contrast, the EMCI group did not show a statistically significant volume reduction relative to the control group (*P*=.20), indicating that substantial OB atrophy may become more evident during later stages of cognitive decline [32,49]. This temporal pattern aligns with the delayed reduction in gamma-band olfactory components (components 2 and 4), suggesting parallel trajectories between structural and functional changes during later stages of the disease. Demographic analyses confirmed minimal influence on our primary outcomes. For OB volume, weak correlations with age (*r*=–0.23; *P*=.05) and education (*r*=0.17; *P*=.15) cannot explain the stage-specific volume reductions observed between groups (*P*<.01). Further analysis of OB volume and frequency-band power revealed a positive correlation between average frequency-band power and OB volume (Figure 11E). Specifically, for gamma bands, the coefficient of determination was 0.2108, with *P*<.001, indicating a strong and statistically significant relationship. In contrast, the correlation observed for the beta bands showed minimal optimization in linear fitting, yielding an *R*^²^ value of 0.0613 (*P*=.05), suggesting a weaker association compared with the gamma bands, although still statistically significant.

## Discussion

### Objective Biomarkers for Early AD Detection

Objective measurement of odorant-mediated olfactory responses has the potential to enable the early diagnosis of AD by accurately predicting clinical neurobehavioral examination scores. In this study, we aimed to determine whether the olfactory response can serve as an early biomarker for AD, enabling us to distinguish between CN, EMCI, LMCI, and DAT groups. Consequently, a significant difference in the average power was observed between individuals who were CN and patients with DAT, which is consistent with the results of the 6-item modified B-SIT. These findings highlight a potential advantage of electrophysiological over behavioral olfactory assessments. While behavioral testing revealed no significant differences between CN and MCI groups, OB recordings showed distinct neural signatures that may reflect subtle disease-related changes. However, given the modest sample size and partial overlap in performance, these results should be considered preliminary. Larger validation studies are warranted to confirm whether OB electrophysiological responses can reliably detect early olfactory dysfunction not captured by behavioral tests. When measuring the electrical signals from the OB across all 4 groups, we observed a decrease in the beta- and gamma-band power and a delay in responses to odor exposure, which correlated with the progression of cognitive dysfunction.

In addition, signals obtained through digitalized olfactory measurements enabled the identification of olfactory response components distributed across various frequencies and time intervals, which allowed for discrimination between the DAT and CN groups at the individual level. The model exhibited outstanding performance in distinguishing among groups: CN (72.22% accuracy), MCI (100% accuracy), and DAT (50% accuracy, 100% precision). These results correlated strongly with the SNSB-II cognitive dysfunction levels and structural characteristics of the brain. Post hoc analyses demonstrated that potential demographic confounders had minimal influence on our findings, with negligible correlations observed between age and cognitive scores (r=–0.16; *P*>.20) and between education and cognitive scores (*r*=0.24; *P*>.05). These findings were further confirmed by formal demographic adjustment, in which the primary electrophysiological predictor retained independent significance for both SNSB-II (*P*=.04) and MMSE (*P*=.009) after controlling for age, sex, and education. While age and education shared some explanatory variance with secondary spectral components, this overlap is expected given the known influence of these factors on cognitive performance and the fact that SNSB-II scores are already standardized for age, sex, and education. Importantly, classification performance did not improve when demographic covariates were included, supporting the independent contribution of electrophysiological features. Future studies with larger, demographically balanced cohorts will be essential to further refine the generalizability and demographic robustness of these electrophysiological biomarkers.

Furthermore, we recorded signals from 6 electrodes using a slightly modified version of a previously reported electrobulbogram to measure the electrical response of the OB. This method has proven to reliably assess OB function in healthy participants, demonstrating high test-retest reliability in previous studies [35]. Notably, patients with PD show a high prevalence of olfactory dysfunction, with approximately 90% of patients experiencing some degree of smell impairment [50]. The high rate of olfactory dysfunction, which is often evident years before the onset of motor symptoms, highlights its potential role as an early indicator of disease. Recently, a noteworthy study presented the electrobulbogram method as an effective tool for detecting PD by distinguishing patients diagnosed with the condition from control participants [37].

### Bidirectional Neurophysiological Insights and mHealth Applications

Olfactory dysfunction is one of the earliest clinical symptoms of AD. It is recognized as both an early indicator and a potential clinical marker of the progression and severity of the disorder [8-10]. In addition to cognitive impairment, approximately 90% of patients with DAT experience loss of olfactory abilities, such as difficulties with olfactory recognition and identification [12]. Sniffing kits, such as UPSIT, are frequently used to examine olfactory dysfunction in patients suspected of having MCI or AD [20-22]. In this study, although the sniffing kit clearly distinguished between healthy individuals and patients with DAT, the differences between healthy and MCI participants were not significant. When electrical signals from the OB were compared across all 4 groups, a decrease in beta- and gamma-band power and delayed responses to odor exposure were observed. Given that the gamma band is primarily associated with processes within the OB [51-55], these observed differences indicated impaired OB processing in DAT due to neuronal loss [10]. Moreover, activity in the beta band is likely generated by top-down projections, which is further supported by the increased latencies and high correlation with cingulum hippocampus diffusivity observed in our data [54,56]. These findings suggest an impairment in the bidirectional interaction between the OB and higher olfactory cortex regions, such as the amygdala and orbitofrontal cortex, which are involved in the processing of various aspects of odor perception. Although a significant reduction in total power was noted between the CN and DAT participants, no significant decrease in total power was found between the CN and MCI groups (early and late) or between the early and late MCI groups. These results suggest a gradual delay in gamma-band synchronization, ultimately leading to a complete loss of olfactory response. This observation is consistent with prior research, indicating that the recognition ability in patients with DAT declines early, whereas the detection threshold is preserved for a relatively longer period [22]. The combined use of TBSS and volumetric analyses offers complementary insights; TBSS enables a detailed investigation of white matter tracts, whereas volumetric quantification assesses regional brain volume changes. This comprehensive structural analysis framework helps map the relationships between functional olfactory components and their underlying neuroanatomical correlations. The observed differential patterns of association suggest distinct neuroanatomical substrates for the beta- and gamma-frequency components of olfactory processing. Our cross-sectional findings show that functional olfactory changes can be present even when volumetric measures remain relatively intact. However, the cross-sectional nature of our study precludes any conclusions about temporal relationships or causality. While we observed functional changes in the presence of preserved structure in some groups, we cannot determine whether electrophysiological changes truly precede volumetric alterations during disease progression. Longitudinal studies are essential to establish these temporal relationships and to confirm whether OB dysfunction represents an early biomarker that emerges before structural brain changes.

In summary, we attempted to digitize the measurement of OB function to differentiate individuals who were CN from patients with EMCI, LMCI, or DAT in a noninvasive manner. Accordingly, we isolated specific olfactory characteristics that significantly differentiate CN and DAT groups, which were successfully regressed against clinical AD diagnostic examinations, demonstrating high levels of sensitivity, specificity, and accuracy. Notably, these olfactory responses are specifically associated with disease characteristics and affected brain regions according to their frequency bands. This finding suggests that the OB, which is responsible for olfactory responses, is pathologically damaged in patients with DAT, resulting in olfactory dysfunction caused not only by the affected brain regions but also by direct biological damage to the OB. Therefore, our results support the potential of this methodology for further development as a tool to facilitate the early diagnosis of AD and provide a robust and objective biomarker for the disease.

### Conclusion

Our study demonstrates that noninvasive digital recordings of OB function can serve as a novel biomarker for continuous and home-based monitoring of cognitive states across the continuum from normal aging to Alzheimer dementia, supporting early detection and timely intervention within an mHealth framework. The electrophysiological biomarkers we identified—particularly the progressive decline in beta-band power and later-stage reduction in gamma-band power—correlate strongly with established clinical and neuroimaging markers of AD progression. These electrophysiological changes may precede structural brain alterations, highlighting their potential value for early detection and longitudinal tracking. As a digital health tool, this technology has potential applications beyond diagnosis, including monitoring disease progression and potentially assessing therapeutic efficacy in clinical trials. Beyond objective signal capture, our wearable olfactory recording system offers distinct practical advantages for mHealth deployment. The device can be fitted in less than 2 minutes, and the entire assessment takes approximately 5‐10 minutes, allowing frequent, home-based measurements with minimal burden on users or caregivers. This approach embodies the mHealth paradigm, where continuous, real-world monitoring of physiological biomarkers enables early detection of cognitive decline and supports timely, personalized interventions. These findings support the development of accessible mHealth technologies for early AD detection and ongoing disease surveillance, addressing the growing need for noninvasive, cost-effective screening tools that can be deployed more widely than expensive neuroimaging techniques or invasive procedures.

## Supplementary material

10.2196/76245 Multimedia Appendix 1 Flowchart of participant recruitment, screening, exclusion, and diagnostic classification. Eligible participants were assigned to the cognitively normal, early mild cognitive impairment, late mild cognitive impairment, or dementia of the Alzheimer type groups based on standardized clinical, neuropsychological, and neuroimaging criteria.

## Abbreviations

AD: Alzheimer disease
Aβ: Amyloid-beta
B-SIT: Brief Smell Identification Test
CN: cognitively normal
CT: computed tomography
DAT: dementia of the Alzheimer type
DTI: diffusion tensor imaging
EMCI: early mild cognitive impairment
FA: fractional anisotropy
LMCI: late mild cognitive impairment
MCI: mild cognitive impairment
mHealth: mobile health
MMSE: Mini-Mental State Examination
MRI: Magnetic Resonance Imaging
NIA-AA: National Institute on Aging and the Alzheimer’s Association
OB: olfactory bulb
PD: Parkinson disease
PET: positron emission tomography
SNSB-II: Seoul Neuropsychological Screening Battery II
SSIM: Structural Similarity Index Matrix
TBSS: tract-based spatial statistics
UPSIT: University of Pennsylvania Smell Identification Test

## References

[1] Shaw LM, Arias J, Blennow K (2018). Appropriate use criteria for lumbar puncture and cerebrospinal fluid testing in the diagnosis of Alzheimer’s disease. Alzheimer’s Dementia.

[2] Hansson O (2021). Biomarkers for neurodegenerative diseases. Nat Med.

[3] Varma VR, Ghosal R, Hillel I (2021). Continuous gait monitoring discriminates community-dwelling mild Alzheimer’s disease from cognitively normal controls. Alzheimers Dement (N Y).

[4] Yan S, Yun X, Liu Q, Hong Z, Chen Y, Zhang S (2025). Advances in gait research related to Alzheimer’s disease. Front Neurol.

[5] Liu DX, Braskie MN, Cavaillès C, Peltz C, Redline S, Yaffe K (2025). Sleep macro-architecture, nocturnal hypoxemia, and Alzheimer’s disease-related MRI patterns among diverse older adults. Alzheimers Dement.

[6] Popp Z, Low S, Igwe A (2024). Shifting from active to passive monitoring of Alzheimer disease: the state of the research. J Am Heart Assoc.

[7] Dieffenderfer J, Brewer A, Noonan MA (2023). A wearable system for continuous monitoring and assessment of speech, gait, and cognitive decline for early diagnosis of ADRD. Annu Int Conf IEEE Eng Med Biol Soc.

[8] Serby M, Larson P, Kalkstein D (1991). The nature and course of olfactory deficits in Alzheimer’s disease. Am J Psychiatry.

[9] Devanand DP, Michaels-Marston KS, Liu X (2000). Olfactory deficits in patients with mild cognitive impairment predict Alzheimer’s disease at follow-up. Am J Psychiatry.

[10] Hummel T, Landis BN, Hüttenbrink KB (2011). Smell and taste disorders. GMS Curr Top Otorhinolaryngol Head Neck Surg.

[11] Murphy C (2019). Olfactory and other sensory impairments in Alzheimer disease. Nat Rev Neurol.

[12] Duff K, McCaffrey RJ, Solomon GS (2002). The Pocket Smell Test: successfully discriminating probable Alzheimer’s dementia from vascular dementia and major depression. J Neuropsychiatry Clin Neurosci.

[13] Doty RL (2017). Olfactory dysfunction in neurodegenerative diseases: is there a common pathological substrate?. Lancet Neurol.

[14] Hawkes C (2006). Olfaction in neurodegenerative disorder. Adv Otorhinolaryngol.

[15] Attems J, Walker L, Jellinger KA (2014). Olfactory bulb involvement in neurodegenerative diseases. Acta Neuropathol.

[16] Velayudhan L (2015). Smell identification function and Alzheimer’s disease: a selective review. Curr Opin Psychiatry.

[17] Attems J, Jellinger KA (2006). Olfactory tau pathology in Alzheimer disease and mild cognitive impairment. Clin Neuropathol.

[18] Sun GH, Raji CA, Maceachern MP, Burke JF (2012). Olfactory identification testing as a predictor of the development of Alzheimer’s dementia: a systematic review. Laryngoscope.

[19] Marin C, Vilas D, Langdon C (2018). Olfactory dysfunction in neurodegenerative diseases. Curr Allergy Asthma Rep.

[20] Silva MdME, Mercer PBS, Witt MCZ, Pessoa RR (2018). Olfactory dysfunction in Alzheimer’s disease systematic review and meta-analysis. Dement Neuropsychol.

[21] Taherkhani S, Moztarzadeh F, Mehdizadeh Seraj J (2015). Iran Smell Identification Test (Iran-SIT): a modified version of the University of Pennsylvania Smell Identification Test (UPSIT) for Iranian population. Chem Percept.

[22] Zou Y ming, Lu D, Liu LP, Zhang H hong, Zhou Y ying (2016). Olfactory dysfunction in Alzheimer’s disease. NDT.

[23] McKhann GM, Knopman DS, Chertkow H (2011). The diagnosis of dementia due to Alzheimer’s disease: recommendations from the National Institute on Aging‐Alzheimer’s Association workgroups on diagnostic guidelines for Alzheimer’s disease. Alzheimers Dement.

[24] Kreisl WC, Jin P, Lee S (2018). Odor identification ability predicts PET amyloid status and memory decline in older adults. J Alzheimers Dis.

[25] Velayudhan L, Gasper A, Pritchard M, Baillon S, Messer C, Proitsi P (2015). Pattern of smell identification impairment in Alzheimer’s disease. J Alzheimers Dis.

[26] Invitto S, Piraino G, Ciccarese V (2018). Potential role of OERP as early marker of mild cognitive impairment. Front Aging Neurosci.

[27] Paitel ER, Samii MR, Nielson KA (2021). A systematic review of cognitive event-related potentials in mild cognitive impairment and Alzheimer’s disease. Behav Brain Res.

[28] Fatemi SN, Aghajan H, Vahabi Z, Afzal A, Sedghizadeh MJ (2022). Behavior of olfactory-related frontal lobe oscillations in Alzheimer’s disease and MCI: a pilot study. Int J Psychophysiol.

[29] Musaeus CS, Engedal K, Høgh P (2018). EEG theta power is an early marker of cognitive decline in dementia due to Alzheimer’s disease. J Alzheimers Dis.

[30] Sedghizadeh MJ, Hojjati H, Ezzatdoost K, Aghajan H, Vahabi Z, Tarighatnia H (2020). Olfactory response as a marker for Alzheimer’s disease: evidence from perceptual and frontal lobe oscillation coherence deficit. PLoS One.

[31] Thomann PA, Dos Santos V, Toro P, Schönknecht P, Essig M, Schröder J (2009). Reduced olfactory bulb and tract volume in early Alzheimer’s disease--a MRI study. Neurobiol Aging.

[32] Jobin B, Boller B, Frasnelli J (2021). Volumetry of olfactory structures in mild cognitive impairment and Alzheimer’s disease: a systematic review and a meta-analysis. Brain Sci.

[33] Thomann PA, Dos Santos V, Seidl U, Toro P, Essig M, Schröder J (2009). MRI-derived atrophy of the olfactory bulb and tract in mild cognitive impairment and Alzheimer’s disease. J Alzheimers Dis.

[34] Vasavada MM, Wang J, Eslinger PJ (2015). Olfactory cortex degeneration in Alzheimer’s disease and mild cognitive impairment. J Alzheimers Dis.

[35] Iravani B, Arshamian A, Ohla K, Wilson DA, Lundström JN (2020). Non-invasive recording from the human olfactory bulb. Nat Commun.

[36] Iravani B, Arshamian A, Lundqvist M, Kay LM, Wilson DA, Lundström JN (2021). Odor identity can be extracted from the reciprocal connectivity between olfactory bulb and piriform cortex in humans. Neuroimage.

[37] Iravani B, Arshamian A, Schaefer M, Svenningsson P, Lundström JN (2021). A non-invasive olfactory bulb measure dissociates Parkinson’s patients from healthy controls and discloses disease duration. NPJ Parkinsons Dis.

[38] Ryu HJ, Yang DW (2023). The Seoul Neuropsychological Screening Battery (SNSB) for comprehensive neuropsychological assessment. Dement Neurocogn Disord.

[39] Kang IW, Beom IG, Cho JY, Son HR (2016). Accuracy of Korean-Mini-Mental Status Examination based on Seoul Neuro-Psychological Screening Battery II results. Korean J Fam Med.

[40] Kang Y, NA DL, Hahn S (1997). A validity study on the Korean Mini-Mental State Examination (K-MMSE) in dementia patients. J Korean Neurol Assoc.

[41] Hua K, Zhang J, Wakana S (2008). Tract probability maps in stereotaxic spaces: analyses of white matter anatomy and tract-specific quantification. Neuroimage.

[42] Smith SM, Nichols TE (2009). Threshold-free cluster enhancement: addressing problems of smoothing, threshold dependence and localisation in cluster inference. Neuroimage.

[43] Fischl B, Salat DH, Busa E (2002). Whole brain segmentation: automated labeling of neuroanatomical structures in the human brain. Neuron.

[44] Doty RL, Marcus A, Lee WW (1996). Development of the 12-item Cross-Cultural Smell Identification Test (CC-SIT). Laryngoscope.

[45] Oostenveld R, Fries P, Maris E, Schoffelen JM (2011). FieldTrip: open source software for advanced analysis of MEG, EEG, and invasive electrophysiological data. Comput Intell Neurosci.

[46] Ezzati A, Katz MJ, Lipton ML, Zimmerman ME, Lipton RB (2016). Hippocampal volume and cingulum bundle fractional anisotropy are independently associated with verbal memory in older adults. Brain Imaging Behav.

[47] Lin YC, Shih YC, Tseng WYI (2014). Cingulum correlates of cognitive functions in patients with mild cognitive impairment and early Alzheimer’s disease: a diffusion spectrum imaging study. Brain Topogr.

[48] Zhang Y, Schuff N, Jahng GH (2007). Diffusion tensor imaging of cingulum fibers in mild cognitive impairment and Alzheimer disease. Neurology (ECronicon).

[49] Servello A, Fioretti A, Gualdi G (2015). Olfactory dysfunction, olfactory bulb volume and Alzheimer’s disease: is there a correlation? A pilot study1. J Alzheimers Dis.

[50] Doty RL (2012). Olfactory dysfunction in Parkinson disease. Nat Rev Neurol.

[51] Yang Q, Zhou G, Noto T (2022). Smell-induced gamma oscillations in human olfactory cortex are required for accurate perception of odor identity. PLoS Biol.

[52] Martin C, Ravel N (2014). Beta and gamma oscillatory activities associated with olfactory memory tasks: different rhythms for different functional networks?. Front Behav Neurosci.

[53] Neville KR, Haberly LB (2003). Beta and gamma oscillations in the olfactory system of the urethane-anesthetized rat. J Neurophysiol.

[54] Frederick DE, Brown A, Brim E, Mehta N, Vujovic M, Kay LM (2016). Gamma and beta oscillations define a sequence of neurocognitive modes present in odor processing. J Neurosci.

[55] Peace ST, Johnson BC, Werth JC (2024). Coherent olfactory bulb gamma oscillations arise from coupling independent columnar oscillators. J Neurophysiol.

[56] Nordén F, Iravani B, Schaefer M (2024). The human olfactory bulb communicates perceived odor valence to the piriform cortex in the gamma band and receives a refined representation back in the beta band. PLoS Biol.

