# Supplementary material for: Noninvasive Electrophysiological Biomarkers of Olfactory Responses Across Cognitive States in Alzheimer Dementia: Cross-Sectional Study
Source: JMIR Mhealth Uhealth. 2026 Jun 23;14:e76245. doi: 10.2196/76245 (PMC13290105; doi:10.2196/76245)

**Trial Protocol**

The olfactory assessment protocol integrated the Brief Smell Identification Test (B-SIT) with simultaneous electroencephalographic (EEG) recording using a six-channel active electrode system (Cyton, OpenBCI, US). EEG data were acquired at 250 Hz to ensure optimal signal resolution. The EEG cap configuration was optimized for detecting olfactory bulb (OB) signals based on the methodology of Iravani et al., utilizing six symmetrically positioned electrodes along the forehead midline, spaced at 5-cm intervals. The adjustable helmet mount ensured precise electrode placement and maintained consistent skin contact throughout the recordings. Signal quality was improved by using bilateral earlobe electrodes as reference points.

The B-SIT administration protocol (Sensonics International) was conducted according to standardized procedures and completed in approximately 10 minutes. The methodology encompassed:

1. Controlled odor release through examiner-activated microcapsules

2. Standardized stimulus presentation at 2-3 cm from the participant's nasal cavity

3. Verbal presentation of four response options for each stimulus

4. Sequential administration of 12 distinct odorants with immediate response recording

The assessment provided a quantitative score based on the number of correct identifications, with a maximum of 12 points. Concurrent EEG recordings captured OB activity dynamics, with data segmented from 1200 ms before stimulus onset to 1800 ms after stimulus presentation. To enhance signal quality, participants kept their eyes closed during the assessment, and trials with excessive artifacts were systematically excluded.

**Eligibility Criteria**

- Participants with major neurological disorders, such as Parkinson's disease, multi-infarct dementia, or other vascular dementias, were excluded.
- Individuals with evidence of infection, infarction, or focal lesions on brain MRI during screening or baseline were excluded, though participants with fewer than five lacunes were eligible.
- A history of major psychiatric disorders, such as schizophrenia, major depression, or bipolar disorder, within the past two years, was an exclusion criterion.
- Uncontrolled sleep disorders, anxiety, or behavioral disorders within three months prior to screening also resulted in exclusion.
- A history of alcohol or drug abuse or dependence within the past two years was exclusionary.
- Participants with malignant tumors diagnosed within three years prior to screening were excluded unless the cancer was in an early and treatable stage.
- Use of medications such as antidepressants, antipsychotics, anxiolytics, or sedative-hypnotics that could interfere with neuropsychological assessments was grounds for exclusion.

**Diagnostic Criteria**

Clinical evaluation consisted of a comprehensive medical history review, neuroimaging analysis, and neuropsychological examination conducted by a qualified physician based on the following criteria.

1. Cognitively normal criteria:

- Absence of subjective memory loss and cognitive decline complaints
- Performance on the delayed recall component of the Seoul Verbal Learning Test at or above "average minus 1.0 standard deviation" on standardized neuropsychological assessments
- Korean Mini-Mental State Examination scores above "mean minus 1.5 standard deviation" according to education and age-adjusted normative data
- No history of cognitive impairment or functional decline in activities of daily living
- Absence of clinical cognitive decline as defined by phases 1 and 2 of the preclinical Alzheimer's disease stages outlined in the National Institute on Aging-Alzheimer's Association research criteria.

2. Early MCI criteria

- Documented history of cognitive impairment, including memory decline, as reported by caregivers and researchers
- Performance on the delayed recall component of the Seoul Verbal Learning Test below "mean minus 1.5 standard deviations" according to education and age-adjusted normative data
- Korean Mini-Mental State Examination scores below "mean minus 1.0 standard deviation" based on education and age-adjusted normative data
- Clinical Dementia Rating score of 0.5 or 1.0
- Meeting the Core clinical criteria as defined by the National Institute on Aging-Alzheimer's Association
- Standardized core score from Seoul Neuropsychological Screening Battery-II over -1 according to education and age-adjusted normative data.

3. Late MCI criteria

- Documented history of cognitive impairment, including memory decline, as reported by caregivers and researchers
- Performance on the delayed recall component of the Seoul Verbal Learning Test below "mean minus 1.5 standard deviations" according to education and age-adjusted normative data
- Korean Mini-Mental State Examination scores below "mean minus 1.0 standard deviation" based on education and age-adjusted normative data
- Clinical Dementia Rating score of 0.5 or 1.0
- Meeting the Core clinical criteria as defined by the National Institute on Aging-Alzheimer's Association
- Standardized core score from Seoul Neuropsychological Screening Battery-II below -1 according to education and age-adjusted normative data.

4.DAT criteria

- Documented history of cognitive impairment, including memory decline, as verified by caregiver and researcher assessments
- Performance on the delayed recall component of the Seoul Verbal Learning Test below "mean minus 1.5 standard deviations" based on education and age-adjusted normative data
- Korean Mini-Mental State Examination scores below "mean minus 1.0 standard deviation" according to education and age-adjusted normative standards
- Clinical Dementia Rating score of 0.5 or 1.0
- Fulfillment of the Core clinical criteria as established by the National Institute on Aging-Alzheimer's Association

**Participants Flowchart**


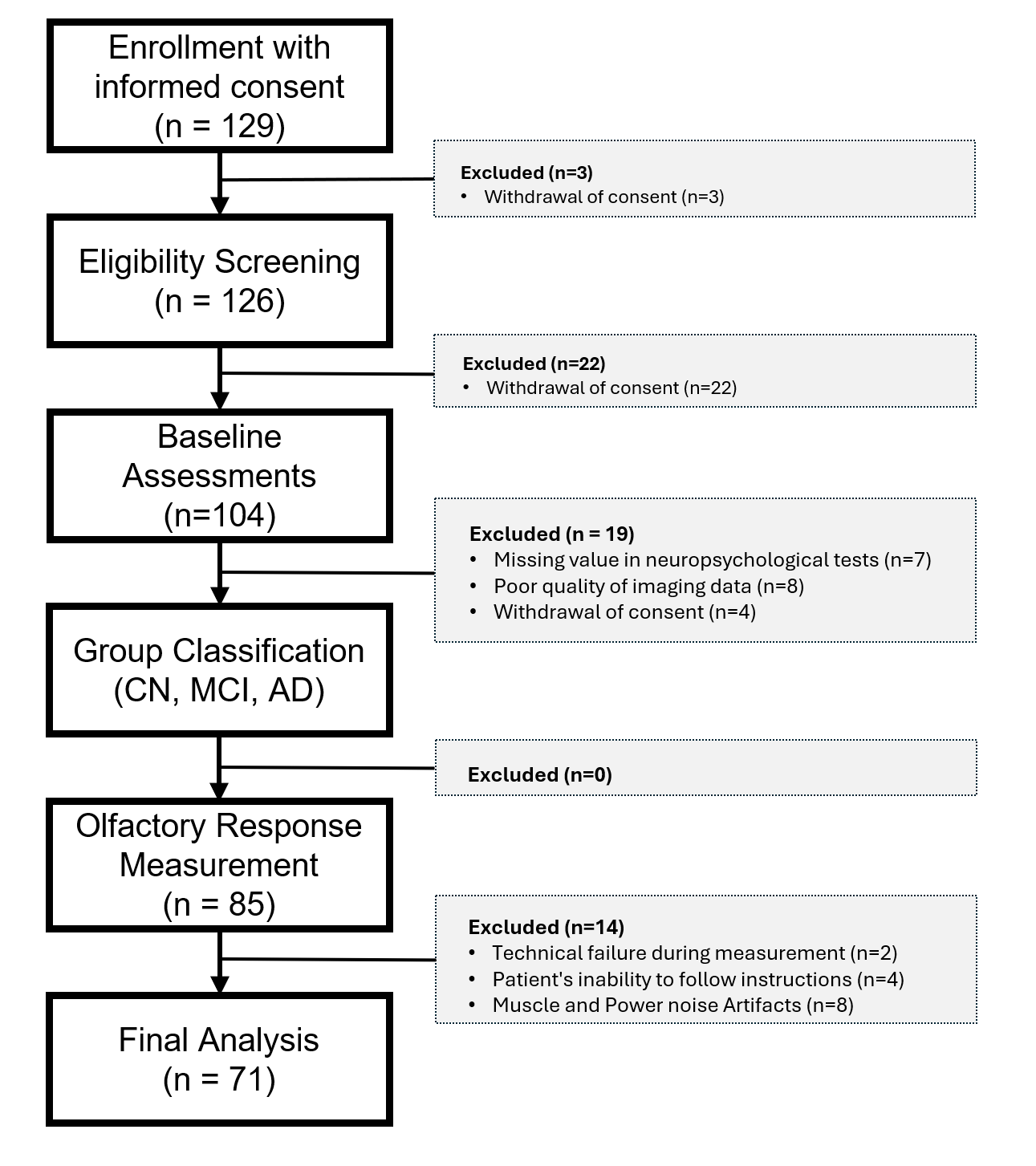

Supplement: Multimedia Appendix 1 [file mhealth-v14-e76245-s001.docx]
